# Perfusion Flow Enhances Osteogenic Gene Expression and the Infiltration of Osteoblasts and Endothelial Cells into Three-Dimensional Calcium Phosphate Scaffolds

**DOI:** 10.1155/2012/915620

**Published:** 2012-09-04

**Authors:** Matthew J. Barron, Jeremy Goldman, Chung-Jui Tsai, Seth W. Donahue

**Affiliations:** ^1^Department of Biomedical Engineering, Michigan Technological University, 1400 Townsend, Houghton, MI 49931, USA; ^2^School of Forestry and Natural Resources and Department of Genetics, The University of Georgia, 111 Riverbend Road, Athens, GA 30602, USA; ^3^Department of Mechanical Engineering, Colorado State University, 1602 Campus Delivery, Fort Collins, CO 80523, USA

## Abstract

Maintaining cellular viability *in vivo* and *in vitro* is a critical issue in three-dimensional bone tissue engineering. While the use of osteoblast/endothelial cell cocultures on three-dimensional constructs has shown promise for increasing *in vivo* vascularization, *in vitro* maintenance of cellular viability remains problematic. This study used perfusion flow to increase osteogenic and angiogenic gene expression, decrease hypoxic gene expression, and increase cell and matrix coverage in osteoblast/endothelial cell co-cultures. Mouse osteoblast-like cells (MC3T3-E1) were cultured alone and in co-culture with mouse microvascular endothelial cells (EOMA) on three-dimensional scaffolds for 1, 2, 7, and 14 days with or without perfusion flow. mRNA levels were determined for several osteogenic, angiogenic, and hypoxia-related genes, and histological analysis was performed. Perfusion flow downregulated hypoxia-related genes (HIF-1**α**, VEGF, and OPN) at early timepoints, upregulated osteogenic genes (ALP and OCN) at 7 days, and downregulated RUNX-2 and VEGF mRNA at 14 days in osteoblast monocultures. Perfusion flow increased cell number, coverage of the scaffold perimeter, and matrix area in the center of scaffolds at 14 days. Additionally, perfusion flow increased the length of endothelial cell aggregations within co-cultures. These suggest perfusion stimulated co-cultures provide a means of increasing osteogenic and angiogenic activity.

## 1. Introduction

While autografts are currently the best available option for bone grafting, the pain and morbidity associated with second surgeries combined with the limited availability of autograft sources highlight the need for alternative grafting materials [1–3]. Three-dimensional bone tissue engineering strategies provide scaffolding for new bone tissue to grow into as well as a cellular source for new tissue growth (osteogenic) [3–6]. However, one of the biggest limitations to overcome is the maintenance of cellular viability within the center of three-dimensional constructs upon *in vivo* implantation [7–11]. Vascularization is critical for successful bone growth and development and is one of the first phases seen during fracture healing [11–16]. Without proper vascularization, bone tissue would face limited growth and hypoxia [13]. As the vasculature provides bone tissue with oxygen, nutrients, growth factors, and the removal of waste, it is not surprising that vascularization is also essential for successful implant integration. Currently, implanted bone grafts rely on the ingrowth of existing vessels from host tissue [7, 9], which can take significant amounts of time, leading to poor vascularization and implant rejection [7]. One solution to this clinical problem is to introduce tissue-engineered bone that is prevascularized by a rudimentary vascular system [7, 9, 10, 17–20]. A common approach to this solution is the co-culture of bone cells and endothelial cells on three-dimensional constructs. 

There has been some success with co-culturing bone cells and endothelial cells to overcome the lack of vasculature in tissue-engineered bone [7–10, 17, 18, 21]. These studies have shown that endothelial cells can form a pre-vascular network during co-culture [9, 10, 17], and endothelial cells enhance osteoblastic activity when both cell types are in direct contact [8, 9, 17, 22, 23]. Additionally, recent work has shown that three-dimensional cocultures will integrate with host tissue *in vivo*, with the formation of an improved vascular network upon implantation [24–26].

Although static cocultures have had some success, it is not known how cocultures respond to a dynamic environment. Perfusion bioreactors deliver culture medium to cell-seeded porous scaffolds through pump-driven fluid flow. As oxygen's poor diffusion capacity largely governs the production of three-dimensional tissue cultures *in vitro* [27, 28], perfusion bioreactors mitigate hypoxic (oxygen-depleted) conditions by providing oxygen delivery to the center of three-dimensional cell-seeded scaffolds, which increases cellular infiltration and viability within three-dimensional scaffolds [27, 29–33]. Perfusion also leads to upregulation in gene expression [29, 31, 34], increases in protein production [30, 33, 35–38], and enhanced calcium deposition [30, 36, 39–41] in osteoblast-seeded scaffolds. Furthermore, fluid flow activates endothelial cells, increasing angiogenic gene expression and the formation of tube-like structures [12, 22, 42–45]. Taken together, these results suggest that the use of a perfusion bioreactor with osteoblast/endothelial cell cocultures may improve osteoblast activity and accelerate the vascularization of tissue-engineered bone by alleviating the hypoxic state of three-dimensional culture.

The goal of this study was to help to elucidate the effects that a dynamic environment has on osteoblast/endothelial cell three-dimensional cocultures. We hypothesized that co-culturing osteoblasts and endothelial cells increases osteogenic and angiogenic gene expressions and decreases hypoxic gene expression in three-dimensional calcium phosphate scaffolds. Furthermore, we hypothesized that perfusion flow increases cell and matrix coverage area throughout three-dimensional scaffolds.

## 2. Materials and Methods

### 2.1. Cell Culture

Mouse osteoblast-like cells (MC3T3-E1 subclone 4, ATCC, Manassas, VA, USA) were cultured in alpha-MEM (Invitrogen, Carlsbad, CA, USA) with 10% FBS (Hyclone, Logan, UT, USA) and 1% pen/strep (Cellgro, Herndon, VA, USA). Mouse microvascular endothelial cells (EC) (EOMA, ATCC, Manassas, VA, USA) were cultured in dulbecco's MEM (Invitrogen, Carlsbad, CA, USA) with 10% FBS (Hyclone, Logan, UT, USA) and 1% pen/strep (Cellgro, Herndon, VA, USA). Once cells reached ~70% confluency, they were trypsinized, resuspended in culture medium and statically seeded onto three-dimensional calcium phosphate scaffolds (BD Biosciences, San Jose, CA, USA) in 25 *μ*L medium (1,000,000 cells/scaffold: 100% osteoblasts or 98% osteoblasts and 2% endothelial cells). The scaffolds have an interconnected porosity of ~60% with an average pore size of 200–400 microns. This is comparable to trabecular bone (50–90% porosity, 500–1500 micron pore size [46]) and within the range suggested to be optimal for bone regeneration (150–650 microns) in porous scaffolds [47]. Cell-seeded scaffolds were incubated for 1 hour to allow cell adhesion, then covered with media (alpha-MEM, 10% FBS, and 1% Pen/Strep), and incubated for 24 hours before experimentation. During 7- and 14-day experimentation, osteoblast differentiation media was used by adding 10 mM beta-glycerol phosphate and 50 mg/mL ascorbic acid. During 1- and 2-day experimentation, nondifferentiation media was used.

### 2.2. Experimental Design

Osteoblast-seeded scaffolds were evaluated after 1 or 2 days of static or perfusion (*n* = 5 per group) culture. Four treatment groups (*n* = 3 per group) were tested at 7- and 14-day time points: two cell populations (osteoblasts alone and osteoblast/endothelial cell co-culture) with two culture methods (static culture and perfusion flow). Static samples were cultured in 24-well plates, while perfusion samples were cultured in bioreactor chambers. All samples remained incubated at 37°C with 5% CO_2_ for the duration of the experiment. Flow was initiated immediately after samples were placed into the bioreactor chambers and continued for the duration of the experiment. After 1, 2, 7, or 14 days of culture (static or perfusion), RNA was isolated from the cell-seeded scaffolds, or the scaffolds were fixed in 10% buffered formalin for histological processing. Gene expression was assessed at all-time points, and histological analysis was performed at the 14 day timepoint.

### 2.3. Perfusion Flow Bioreactor

**Figure 1:**
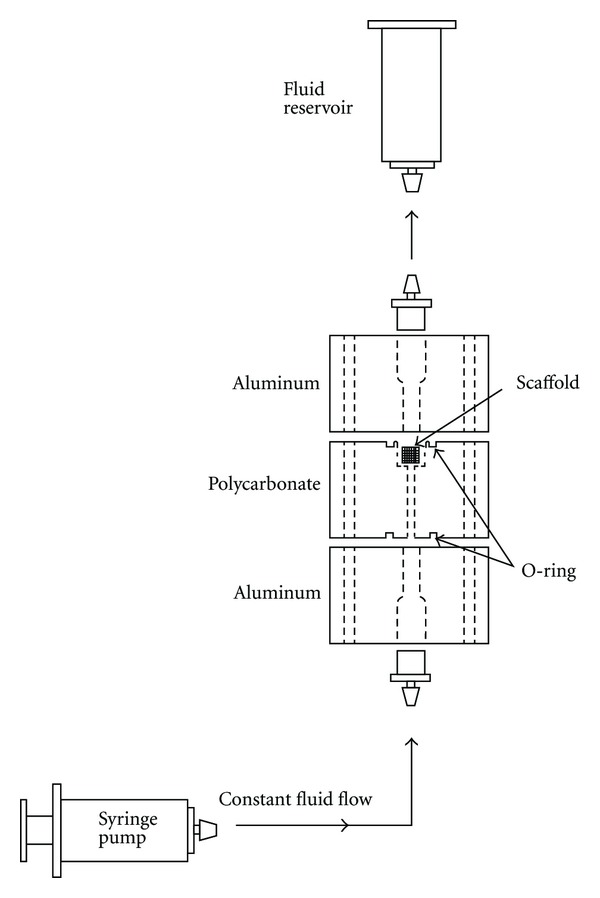
Bioreactor setup for perfusion incubation. The syringe pump delivered fluid at a rate of 0.075 mL/min.

A custom perfusion bioreactor was used to deliver culture media to the cell-seeded calcium phosphate scaffolds. Each bioreactor chamber consists of a top and bottom aluminum block, between which a polycarbonate block is secured (Figure 1). A 6 mm hole was drilled in the polycarbonate block to fit the scaffolds (5 mm diameter; 3.5 mm height). Grooves were machined around the holes on each side of the polycarbonate block to fit a #11 Viton O-ring (Allorings, Hampton Falls, NH, USA). A 3 mm barbed pipe connector was threaded into both the top and bottom aluminum blocks and connected to 3 mm silicone tubing (Harvard Apparatus, Holliston, MA, USA). The silicone tubing is permeable to CO_2_ and O_2_, permitting adequate gas exchange. Tubing connected the top of the chamber to a 140 mL syringe fluid reservoir and the bottom of the chamber to syringe pump (Harvard Apparatus). The pump delivered media at a rate of 0.075 mL/min, with flow being reversed every 24 hours. This flow rate produced an estimated shear stress of <0.001 Pa [48]. Media was replenished every third day.

### 2.4. RNA Isolation and RT-PCR

After 1, 2, 7, and 14 days of culture, cell-seeded scaffolds were placed in 2 mL tubes and crushed in SV Lysis Buffer with a glass rod. After lysis, dilution buffer was added along with 200 mM phosphate buffer to elute the RNA. RNA was isolated from cells using the Promega SV total RNA Isolation kit according to manufacturer's instructions. RNA concentration and quality were determined using spectrophotometer (Nanodrop ND-1000 spectrophotometer, Nanodrop Technologies, Rockland, DE, USA) readings at 260, 230, and 280 nm. Gel electrophoresis was used to verify RNA integrity. RNA was reverse transcribed into cDNA using a reaction mix consisting of Superscript II reverse transcriptase (Invitrogen, Calrsbad, CA, USA), 1x first strand buffer (Invitrogen), 800 *μ*M dNTPs (Promega), Rnase out recombinant ribonuclease inhibitor (Invitrogen), 48 mM dithiothreitol (Invitrogen) and 0.5 *μ*g Oligo(dT)_12-18_ pimer at 42°C for 20 minutes, 50°C for 10 minutes, and 42°C for 1 hour in the Mastercycler Gradient Thermocycler (Eppendorf, Westbury, NY, USA). cDNA was then used for real-time PCR for the genes of interest and the housekeeping genes (Table 1).

All reactions were performed in the StepOnePlus real-time PCR system (Applied Biosystems) under the following cycle parameters: hot start at 95°C for 15 minutes followed by 40 cycles of 95°C for 15 seconds, 60°C for 30 seconds, and 72°C for 15 seconds. Threshold fluorescence was set to 1500 dR and the *C*_*t*_ value was determined for all reactions. *C*_*t*_ values were used to determine the relative up-or downregulation for each gene using the relative standard curve method and normalizing to housekeeping genes that do not change between treatments [49].

### 2.5. Histological Analysis

**Figure 2:**
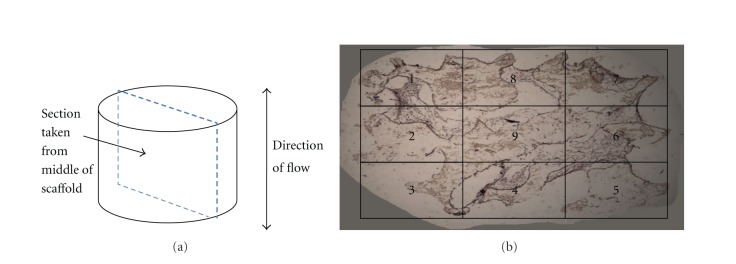
(a) Sections were removed from the middle of the scaffold parallel to the direction of flow. Nine sample regions from each section were used to quantify cell number, surface coverage, and cell/matrix area coverage. (b) Regions 1–8 are combined and are referred to as the scaffold perimeter. Region 9 is referred to as the scaffold center.

Following fixation, 14-day samples were placed in cassettes, covered in OCT freeze medium, and placed under vacuum for 48 hours to remove air bubbles. Samples were then removed, flash frozen, and sectioned on a soft tissue microtome (Microm International, Waldorf, Germany) at a thickness of 10 microns. Sections were taken from mid-way through the scaffold running parallel to the direction of perfusion flow (Figure 2). Sections were decalcified in 0.5% EDTA for 20 minutes. After washing with PBS, biotin-conjugated antibody (1 : 250 antimouse CD31—endothelial cell-specific) was added and samples were incubated for 1 hour. Sections were again washed and the secondary antibody (1 : 500, Alexa Fluor 755) was added and samples were incubated for 45 minutes. Following a brief rinse, sections were also stained with hematoxylin and eosin. Prior to fluorescent microscopy, DAPI was added to the sections to identify cell nuclei. Images were captured for the entire section from each sample at 100x total magnification using both brightfield and fluorescent microscopy. Nine images were taken in total, three from the upper, middle, and lower thirds of each section (Figure 2). Fluorescent images were used to distinguish between cell types, as DAPI identifies all cell nuclei and CD31 stains membrane receptors on endothelial cells. Brightfield images were used to determine total cell number, cell surface coverage, and cell/matrix area coverage.

**Figure 3:**
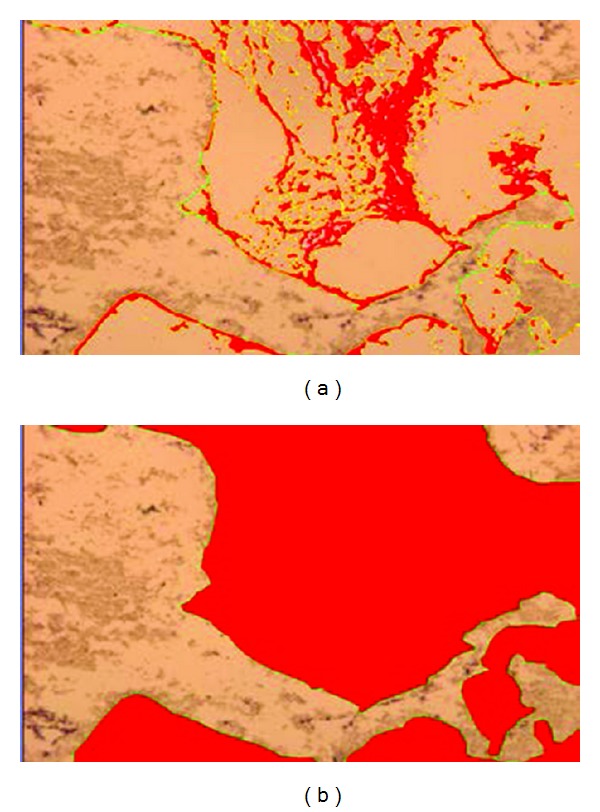
40x magnification. To determine cell/matrix area coverage, a ratio was calculated between (a) area of cell/matrix coverage and (b) total area of pore spaces. The area in red was used for measurement and was determined by setting a threshold value in Bioquant.

Cell number, cell surface coverage, and cell/matrix area coverage were determined around the periphery of the scaffold as well as in the scaffold center, allowing for the assessment of whether perfusion flow is more effective in areas of poor diffusion. For the scaffold periphery, regions 1–8 were combined. For the scaffold center, only the center image (region 9) was used for measurement (Figure 2). Cell number was determined by counting the total cell number in each region within a section. In the scaffold periphery, cell number was averaged for all 8 regions (regions 1–8, Figure 2) for comparison with the scaffold center (region 9, Figure 2). Cell surface coverage was calculated as the ratio of the length of cell-covered scaffold surface to the total length of scaffold surface using Bioquant (Bioquant Osteo, Nashville, TN, USA) software (Figure 2). Cell/matrix area coverage was calculated as the ratio of cell and matrix area within pore spaces to total pore space area (Figure 3).

**Figure 4:**
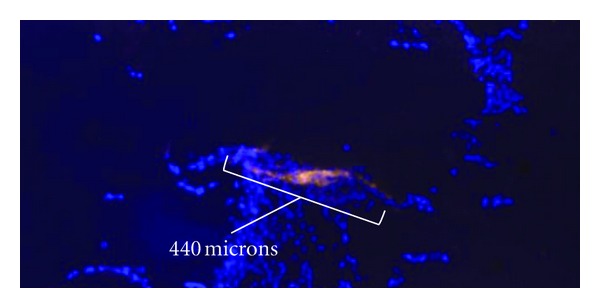
40x magnification. Fluorescent image of osteoblast/endothelial cell cocultures—dapi stained nuclei (blue) and CD31 antibody (bright orange) for endothelial cells. White bracket designates aggregate length measurement.

To measure the length of endothelial cell aggregations, fluorescent microscopy was used to distinguish between cell types, and Bioquant was used to determine endothelial cell aggregate length based on standard scale measurements (Figure 4). The number of endothelial cell aggregates was small due to the low ratio of endothelial cells seeded initially. Thus, additional sections were taken from 25% and 75% of the way through each sample for additional measurements of aggregation length.

### 2.6. Statistics

A one-factor ANOVA was used to compare mRNA levels in the 1- and 2-day osteoblast cultures between static and perfusion incubation. A two-factor ANOVA (cell population and incubation method) was used for the 7- and 14-day cocultures. Both cell population and incubation methods had model effects for some genes, so one-factor ANOVAs were performed within both cultures alone to compare incubation methods, and within static incubation to compare cell populations (osteoblasts alone versus co-culture). 

A two-factor ANOVA (cell population and culture method) was used to assess the histological outcome variables. No significance was associated with cell population (osteoblast versus co-culture) for cell number (*P* = 0.94), surface coverage (*P* = 0.64), or cell/matrix area coverage (*P* = 0.43), and no interactions existed between factors. Thus, values were combined for osteoblast and co-culture treatments. A one-factor ANOVA was then used to compare cell number, surface coverage, and cell/matrix area coverage between static culture and perfusion flow in both the scaffold perimeter (regions 1–8 combined) and the scaffold center (region 9) (Figure 2). A one-way ANOVA was used to compare endothelial cell aggregation lengths between static and perfusion cultures. JMP IN 5.1 statistical software package (SAS, Cary, NC, USA) was used for all statistical analyses.

## 3. Results

### 3.1. Gene Expression (1-and 2-Day Osteoblast Cultures)

After 24 hours of flow, OPN (a marker of both bone development and the inflammatory response) was downregulated (*P* = 0.03) by 10% as was VEGF (an angiogenic growth factor) by 48% (*P* = 0.02). In contrast, COX-2, another marker of both bone development and the inflammatory response, was upregulated (*P* = 0.02) by perfusion flow by 67% (Table 2).

After 48 hours of perfusion flow, while the same trend existed for OPN, VEGF, and COX-2 at 24 hours, the responses were not statistically significant (*P* > 0.20). However, HIF-1*α*, the major transcription factor driving the hypoxic response, was downregulated (0.02) by 55% (Table 3).

Runx-2, Col1, M-CSF, bFGF, and GBE-1 were not significantly different between perfusion and static treatments at either time points (*P* > 0.20).

### 3.2. Gene Expression (7-and 14-Day Cocultures)

After 7 days of culture, mRNA levels of both ALP and OCN were upregulated (over static culture) by 142% (*P* = 0.001) and 819% (*P* = 0.02), respectively, when osteoblast cultures alone was exposed to perfusion flow (Table 3). Similarly, ALP and OCN were upregulated (over static culture) by 143% (*P* = 0.04) and 1072% (*P* = 0.07), respectively, when cocultures were exposed to perfusion flow (Table 4), although OCN only approached significance. This was expected, as ALP and OCN, both markers of osteoblast differentiation, have been shown to be increased in three-dimensional scaffolds exposed to perfusion culture [29, 30, 36, 40, 50]. RUNX-2, OPN, Col1, and VEGF did not change after 7 days of culture (*P* > 0.2).

After 14 days of culture, mRNA levels of RUNX-2 and VEGF were downregulated by 42% (*P* = 0.01) and 85% (*P* < 0.0001), respectively, when osteoblast cultures were exposed to perfusion flow (Table 3). Similarly, perfusion flow downregulated mRNA levels of RUNX-2 by 55% (*P* = 0.01) when cocultures were exposed to perfusion flow (Table 4). These results were unexpected as both genes have been shown to be upregulated as osteoblasts differentiate, and 7-day mRNA levels indicated an increase in differentiation-related genes (ALP and OCN). 

Co-culturing endothelial cells with osteoblasts (static and perfusion combined) caused changes in only one gene at both time points. After 7 and 14 days of static culture, RUNX-2 was downregulated in cocultures by 57% (*P* = 0.01) and 26% (*P* = 0.04) compared to osteoblast alone. No other changes were evident (*P* > 0.2).

### 3.3. Histological Analysis: Cell Number, Surface Coverage, and Cell Area Coverage

**Figure 5:**
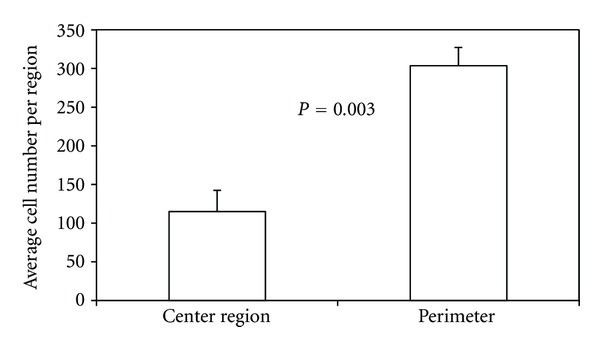
Cell number is lower in the middle region of three-dimensional scaffolds cultured statically for 14 days. Periphery is mean value of the 8 peripheral regions.

**Figure 6:**
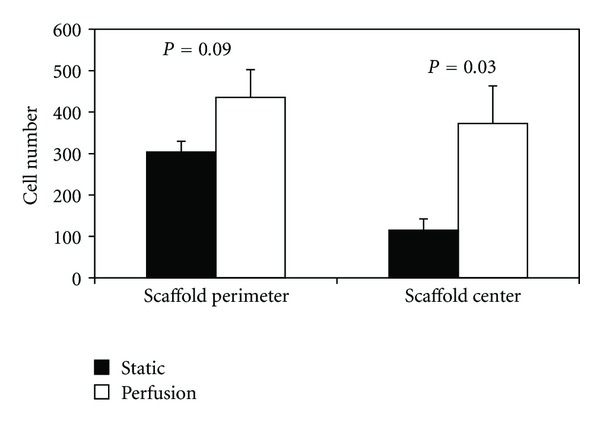
Cell number is increased in three-dimensional scaffolds when exposed to perfusion flow for 14 days. Periphery is mean value of the 8 peripheral regions.

**Figure 7:**
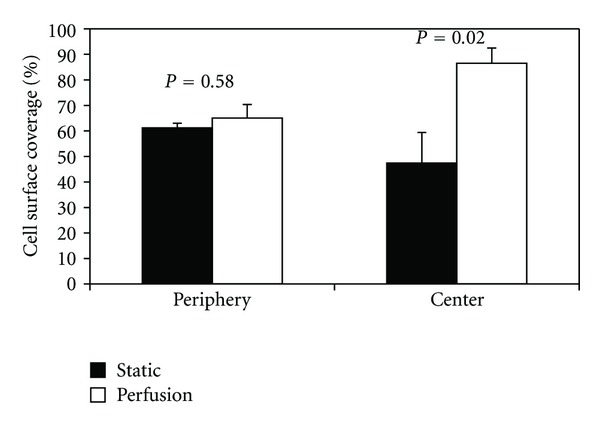
Surface coverage is increased in three-dimensional scaffolds when exposed to perfusion flow for 14 days. Cell surface coverage was calculated as a % of the length of cell-covered scaffold surface to the total length of scaffold surface. Periphery is the mean value of the 8 peripheral regions.

**Figure 8:**
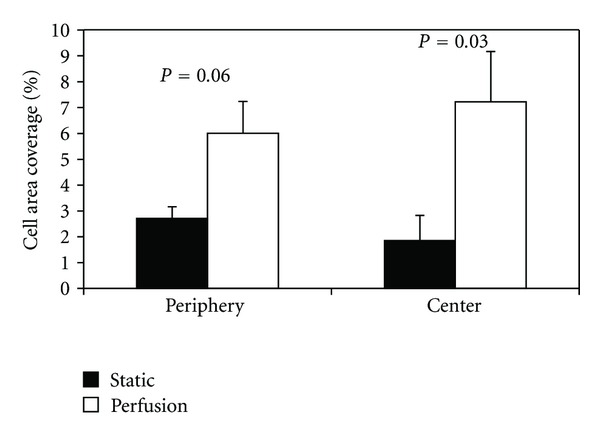
Cell/matrix area coverage is increased in three-dimensional scaffolds when exposed to perfusion flow for 14 days. Cell/matrix area coverage was calculated as a percentage of cell and matrix area within pore spaces to total pore space area. Periphery is the mean value of the 8 peripheral regions.

Under static culture, cell number was 60% lower (*P* = 0.003) in the scaffold center (region 9) as compared to the scaffolds' periphery (regions 1–8) (Figure 5). Cell surface coverage and cell/matrix area coverage were not significantly different between the scaffold center and periphery (*P* > 0.45). Around the scaffold periphery, perfusion flow had no effect on surface coverage (*P* = 0.58). However, perfusion-induced increases in cell number (35%) and cell/matrix area coverage (140%) approached significance (*P* = 0.09 and *P* = 0.06, resp.). Furthermore, in the scaffold center, perfusion flow increased cell number (*P* = 0.03), surface coverage (*P* = 0.02), and cell/matrix area coverage (*P* = 0.03) by 220%, 84%, and 280%, respectively, compared to static culture (Figures 6, 7, and 8). These findings suggest the scaffolds' centers are more responsive to perfusion flow than the periphery.

### 3.4. Histological Analysis: Endothelial Cell Aggregate Length

Endothelial cells were found along the surfaces of the scaffold rather than within the matrix formed by osteoblasts inside the scaffold pores. Additionally, endothelial cell aggregations were found in all 9 sample regions of the scaffold and did not appear to be more highly concentrated in any particular region of the scaffolds (periphery versus center). Furthermore, endothelial cells were not found as individual cells, but rather as aggregations of many cells. This was true throughout all regions. Aggregations seemed to be oriented in all directions on the scaffold surface and did not tend to be aligned in the direction of fluid flow.

**Figure 9:**
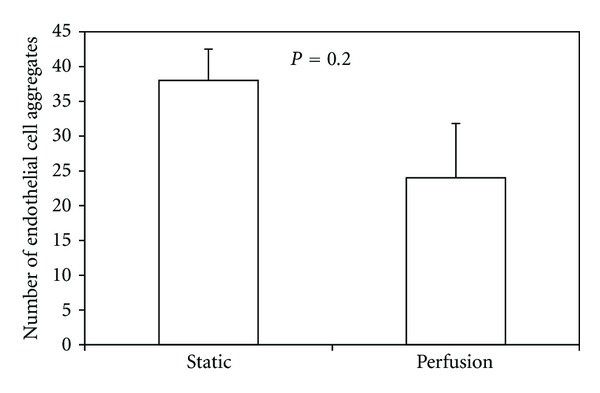
The average number of endothelial cell aggregates did not change in three-dimensional scaffolds subjected to perfusion flow compared to static conditions.

**Figure 10:**
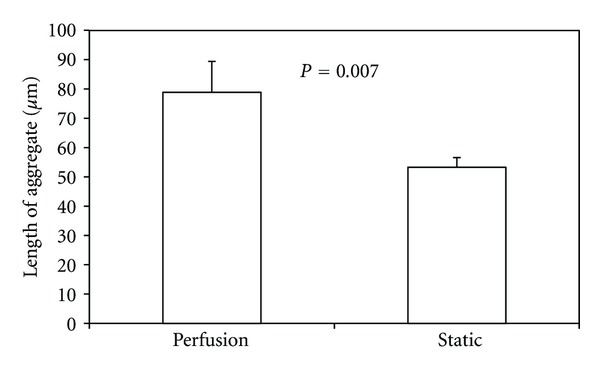
The average length of endothelial cell aggregates was increased in cell-seeded three-dimensional scaffolds and exposed to perfusion flow (*n* = 72 aggregates) compared to static conditions (*n* = 114 aggregates).

**Figure 11:**
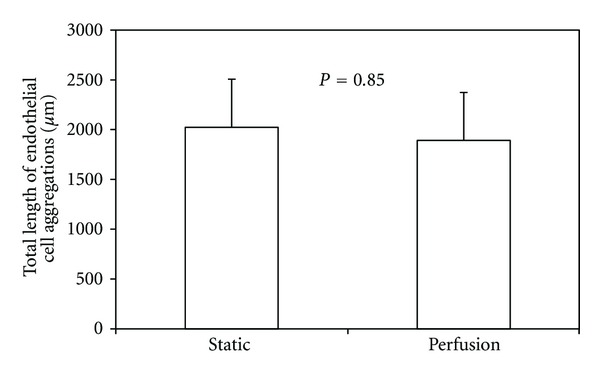
Total length of endothelial cell aggregations does not change with perfusion flow.

There was no difference (*P* = 0.31) in the aggregate number between the scaffold periphery and the scaffold center. Furthermore, there was no difference (*P* = 0.20) in aggregate number between static culture and perfusion flow (Figure 9). However, the average endothelial cell aggregate length increased by almost 50% (*P* = 0.007) in perfusion flow samples compared to static samples (Figure 10). There was no difference in total endothelial cell aggregate length in static culture versus perfusion flow (Figure 11).

## 4. Discussion

The ultimate goal of tissue engineering is to produce high-quality bone tissue *in vitro* to be used as a clinical alternative to autografting. Three-dimensional structures cultured *in vitro* encounter limited vascular invasion upon implantation (7–10). Here, we co-cultured endothelial cells and osteoblasts to develop a method which might improve vascularization following implantation of tissue-engineered bone constructs *in vivo*. Specifically, we used perfusion flow to increase the length of endothelial cell aggregations within cocultures, osteoblast-specific gene expression, and the cell number, cell coverage of the scaffold perimeter, and matrix area in the scaffold pores in the center of the scaffold.

Although there is no existing literature on the effects of perfusion flow on cocultures, there have been studies which have achieved vascular-like network formation in static osteoblast/endothelial cell cocultures [10]. While we showed that perfusion flow can increase the length of endothelial cell aggregations, we did not provide evidence of the formation of vascular-like networks among endothelial cell populations. To form a functioning vascular system, it will be advantageous for endothelial cell aggregates to eventually connect with one another. By increasing aggregate length, perfusion flow may make it more likely for neighboring endothelial cell aggregates to connect and communicate effectively. The low ratio (2%) of endothelial cell seeding may not have allowed for communication between populations of endothelial cells, which is necessary for network formation. Alternatively, while the scaffold pores are interconnected, the porosity of the 3D scaffolds used here is relatively low in comparison to scaffolds used in previous studies. Increasing porosity and maintaining the pore interconnectedness would likely increase aggregate communication. In the future, it may be necessary to increase the ratio of endothelial cells and/or the porosity of the scaffolds to increase interaction between aggregations, enhancing network formation. 

In static culture, the center region of the scaffolds had lower cell number than in perimeter regions. Perfusion flow successfully increased cell number as well as cell coverage of the scaffold perimeter and matrix area in the scaffold pores in the center of the scaffold. These three responses were not seen along the scaffold periphery. This amplified response within the center of the scaffold may be explained by considering diffusional limitations associated with three-dimensional tissue engineering [4, 27, 28, 51]. Three-dimensional cell-seeded constructs have reduced cell numbers and cellular activity towards their centers due to poor oxygen and nutrient delivery. Perfusion flow has been used to mitigate these limitations by increasing oxygen and nutrient delivery [27], consequently increasing cell number and matrix production in the center of three-dimensional constructs. These results may be attributed to the interconnected pore structure of 3D scaffolds as the medium allows for communication between cells via paracrine signaling. It is well known that successful tissue-engineered scaffolds require a high degree of pore interconnectedness. Here, we provide evidence that perfusion flow affected the scaffolds' center to a greater degree than it affected the scaffolds' periphery, suggesting that perfusion bioreactors capitalize on a scaffold's porous structure. 

Previous research has shown that perfusion bioreactors reduce hypoxia in three-dimensional scaffolds [27, 30, 36]. Hypoxia elicits a well-documented response from mesenchymal stem cells and osteoblasts, including the upregulation of vascular endothelial growth factor (VEGF), a major mediator of both angiogenesis and hypoxia [52, 53]. We found that perfusion flow reduces the expression of VEGF in three-dimensional osteoblast monocultures (1- and 2-day cultures) and in three-dimensional osteoblast monocultures (14-day culture). Perfusion also reduced HIF-1*α*, a major regulator of hypoxia, in three-dimensional osteoblast monocultures (2-day culture). In addition, cell number, cell coverage of the scaffold perimeter, and matrix area in the scaffold pores are all increased in the scaffold center with perfusion flow (14-day culture). Thus, hypoxia-specific gene regulation supports histological evidence of increased cellular activity, suggesting that perfusion flow mitigates a hypoxic state by providing adequate oxygen delivery to the center of three-dimensional constructs. 

In addition to histological and hypoxia-specific evidence, our results also indicate that perfusion flow increases bone specific gene expression of ALP and OCN at seven days in both osteoblast monocultures and osteoblast/endothelial cell cocultures, which is consistent with previous reports [29, 30, 36]. However, in contrast to these results, RUNX-2 was reduced in both osteoblast and cocultures with perfusion flow after 14 days of culture. Osteoblast differentiation genes tend to follow a well-defined pattern of expression throughout the process of maturation. ALP, OCN, and OPN are all upregulated transiently, tend to peak after 2-3 weeks, and subsequently drop in expression as osteoblasts differentiate into osteocytes [54, 55]. As RUNX-2 is a transcription factor responsible for osteoblast differentiation, it is not surprising that its expression precedes osteoblast-specific genes. A possible explanation for the decrease in RUNX-2 expression with perfusion flow is that it has reached its peak expression earlier in perfused samples and has begun its decline in expression at 14 days. 

The increase in endothelial cell aggregation length highlights the potential for perfusion bioreactors in three-dimensional bone tissue engineering. By increasing the *in vitro* activity of ECs in bone tissue constructs, perfusion flow could serve to speed up the process of host acceptance in future clinical applications. A higher quality vascular-like network *in vitro* will have a greater likelihood of attaining successful anastomosis with host vasculature *in vivo*. Even if functional anastomosis is not fully achieved, perfusion-induced angiogenic activity could potentiate paracrine signaling between cells within the scaffold and host vasculature, advancing vascular infiltration. These results suggest the potential for perfusion bioreactors to improve *in vitro* co-culturing to maximize the quality and effectiveness of vascularized bone tissue cultures.

## Figures and Tables

**Table 1:** Primer sequences and roles for genes measured in three-dimensional perfusion studies.

Gene name	Function	Primer sequence
Osteocalcin	Osteoblast differentiation	F: 5′-GAGTCTGACAAAGCCTTCATGTCC-3′;
		R: 5′-TGATAGCTCGTCACAAGCAGGGTTA-3′

Osteopontin	Osteoblast differentiation	F: 5′-CAGCTGGATGAACCAAGTCTGGAA-3′;
		R: 5′-ACTAGCTTGTCCTTGTGGCTGTGA-3′

Alkaline phosphatase	Osteoblast differentiation	F: 5′-GCCCTCTCCAAGACATATA-3′;
		R: 5′-CCATGATCACGTCGATATCC-3′

RUNX-2	Osteoblast differentiation	F: 5′-AGAGTCAGATTACAGATCCCAGGC-3′;
		R: 5′-GTCAGAGGTGGCAGTGTCATCAT-3′

Collagen type 1	Matrix protein	F: 5′-TGGTTTGGAGAGAGCATGACCGAT-3′;
		R: 5′-TGTAGGCTACGCTGTTCTTGCAGT-3′

Vascular endothelial growth factor A	Angiogenesis	F: 5′-ACAGAAGGAGAGCAGAAGTCCCAT-3′;
		R: 5′-ATGTGCTGGCTTTGGTGAGGTTTG-3′

Basic fibroblast growth factor 2	Angiogenesis	F: 5′-AGCGGCTCTACTGCAAGAAC-3′;
		R 5′-TGGCACACACTCCCTTGATA-3′

Macrophage colony stimulating factor	Angiogenesis	F: 5′-ATGGACACCTGAAGGTCCTG-3′;
		R: 5′-GCTGGAGAGGAGTCTCATGG-3′

COX-2	Inflammatory/osteogenic	F: 5′-TCAATACTGGAAGCCGAGCACCTT-3′;
		R: 5′-GCACTTGCATTGATGGTGGCTGTT-3′

HIF-1*α*	Hypoxia	F: 5′-AAACTTCTGGATGCCGGTGGTCTA-3′;
		R: 5′-TCTCACTGGGCCATTTCTGTGTGT-3′

GBE-1	Hypoxia	F: 5′-GCAGGTATAAGAAGTTTAGCCAGG-3′;
		R: 5′-GAGAAAATGGATTCCAACCACTGAA-3′

MIF	Hypoxia	F: 5′-CGCACAGTACATCGCAGTG-3′;
		R: 5′-CAGCGGTGCAGGTAAGTG-3′

Cyclophilin	Housekeeping	F: 5′-TCATGTGCCAGGGTGGTGACTTTA-3′;
		R: 5′-ATGCTTGCCATCCAGCCATTCAGT-3′

Beta-actin	Housekeeping	F: 5′-ATCACTATTGGCAACGAGCGGTTC-3′;
		R: 5′-TCTCCTTCTGCATCCTGTCAGCAA-3′

Ubiquitin	Housekeeping	F: 5′-CGTCGAGCCCAGTGTTACCACCAAGAAGG-3′;
		R: 5′-CCCCCATCACACCCAAGAACAAGCACAAG-3′

**Table 2:** Changes in mRNA levels with perfusion flow (osteoblast cultures) at 1 and 2 days.

Gene	7-day fold change	*P* value	14-day fold change	*P* value
VEGF	↓48%	0.02	—	0.32
OPN	↓10%	0.03	—	0.14
COX-2	↑67%	0.02	—	0.25
HIF-l*α*	—	—	↓45%	0.02

**Table 3:** Changes in mRNA levels with perfusion flow at 7- and 14-day samples (osteoblast cultures).

Gene	7-day fold change	*P* value	14-day fold change	*P* value
ALP	↑142%	0.001	—	0.11
OCN	↑819%	0.02	—	0.91
RUNX-2	—	0.26	↓65%	0.01
VEGF	—	0.55	↓85%	<0.0001

**Table 4:** Changes in mRNA levels with perfusion flow at 7- and 14-day samples (co-cultures).

Gene	7-day fold change	*P* value	14-day fold change	*P* value
ALP	↑143%	0.04	—	0.19
OCN	↑1072%	0.07	—	0.35
RUNX-2	—	0.73	↓55%	0.01
